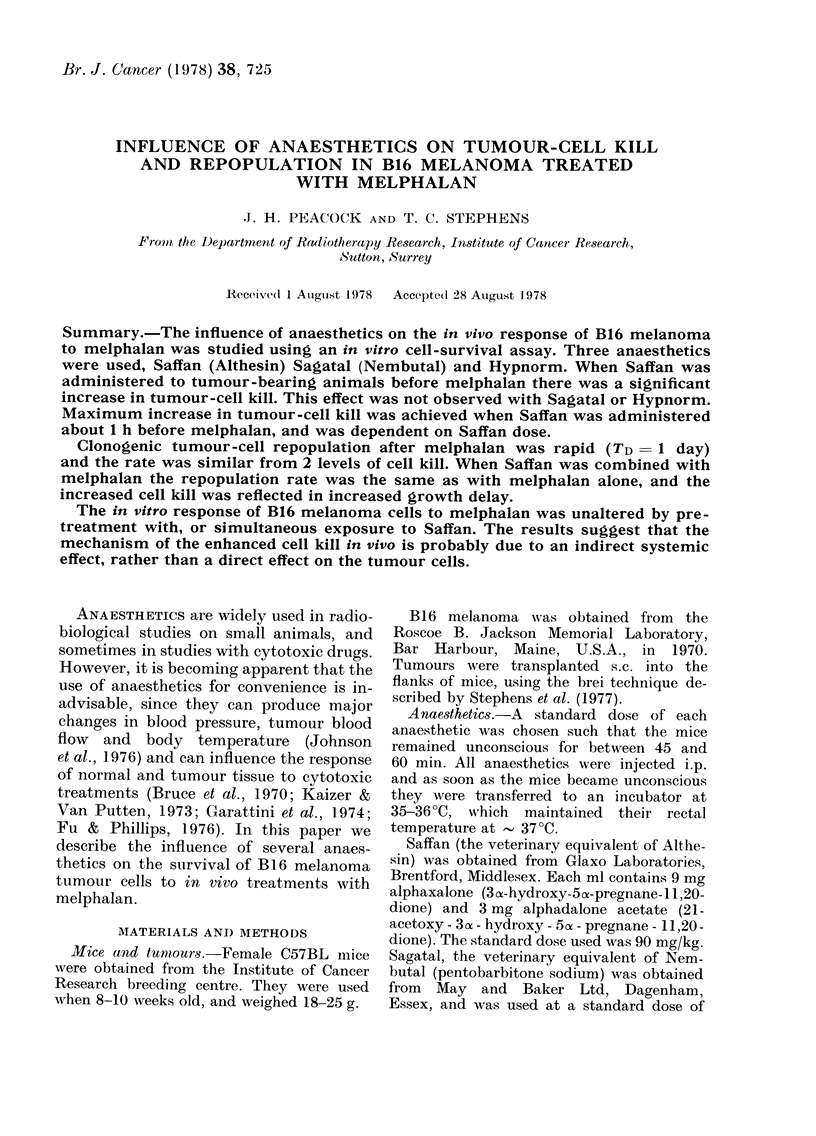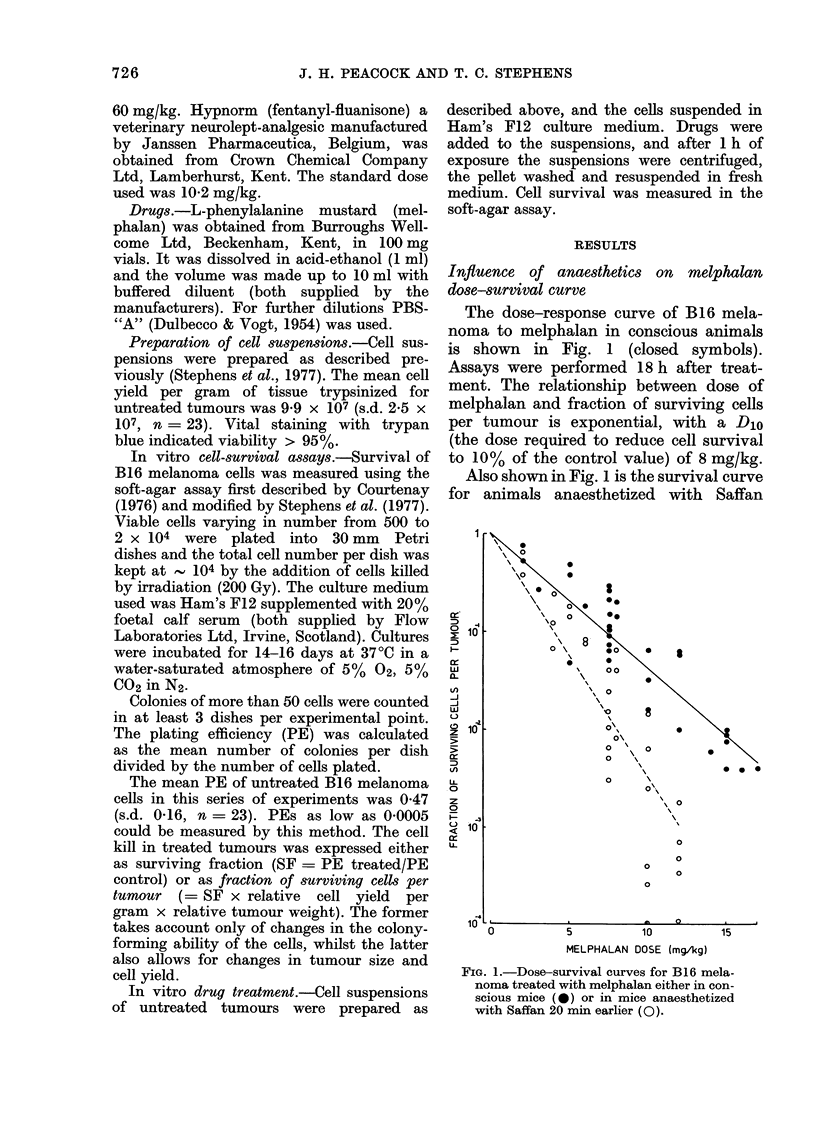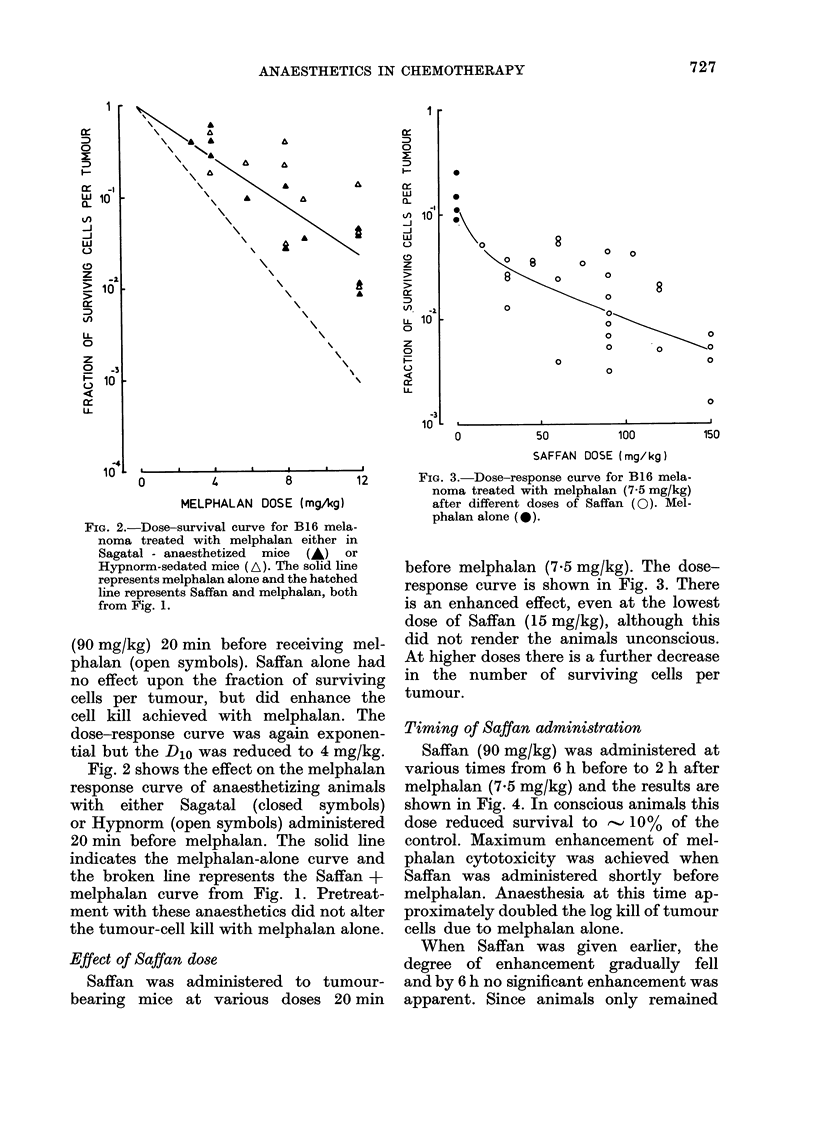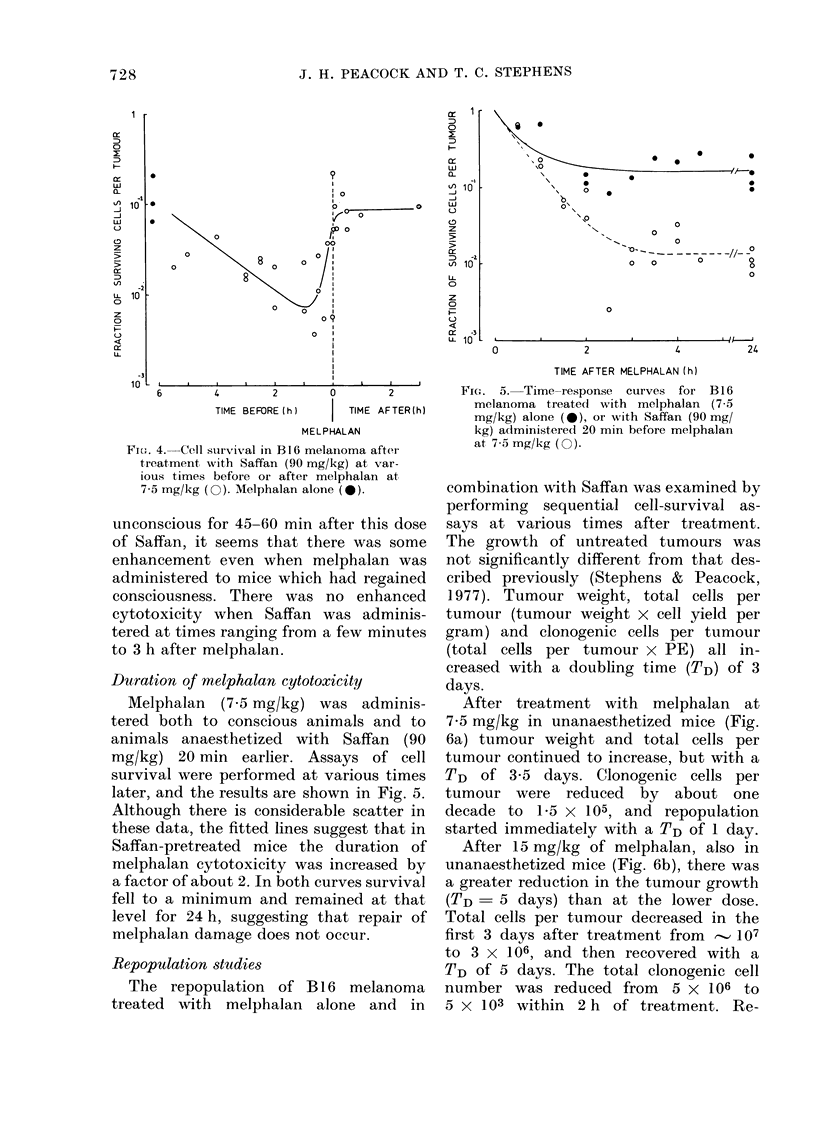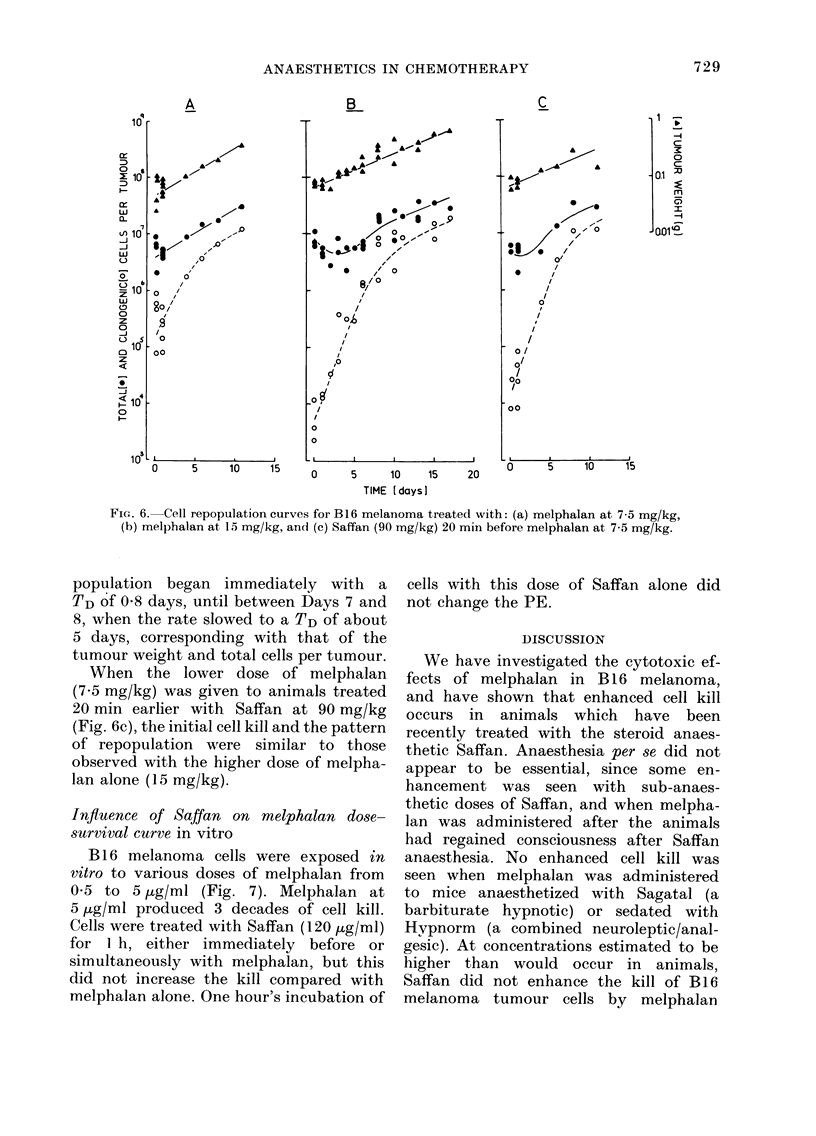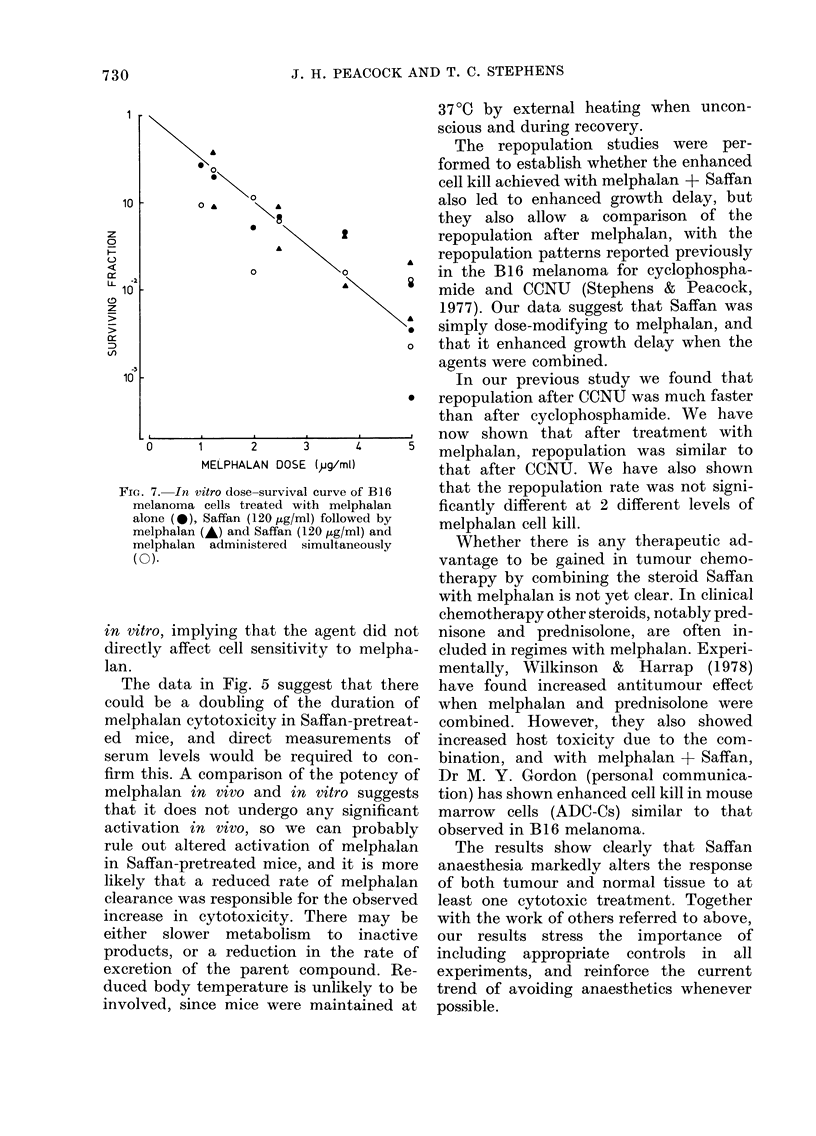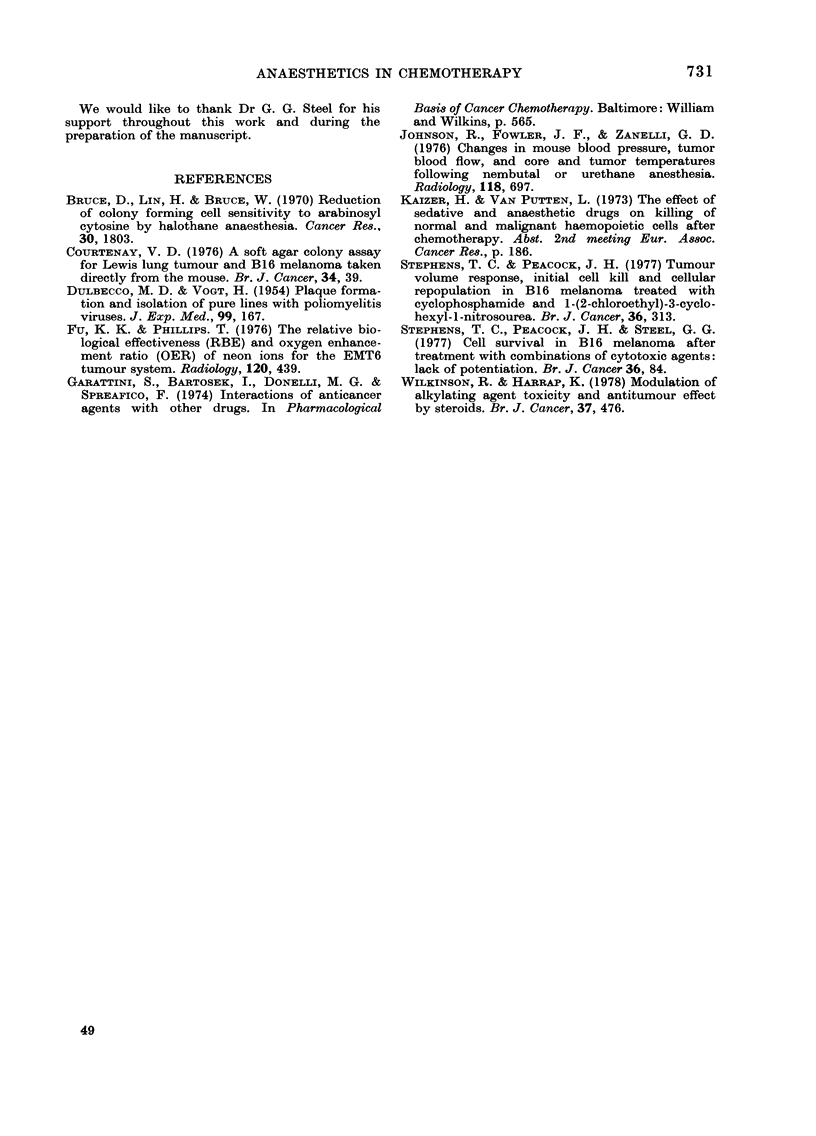# Influence of anaesthetics on tumour-cell kill and repopulation in B16 melanoma treated with melphalan.

**DOI:** 10.1038/bjc.1978.279

**Published:** 1978-12

**Authors:** J. H. Peacock, T. C. Stephens

## Abstract

The influence of anaesthetics on the in vivo response of B16 melanoma to melphalan was studied using an in vitro cell-survival assay. Three anaesthetics were used, Saffan (Althesin) Sagatal (Nembutal) and Hypnorm. When Saffan was administered to tumour-bearing animals before melphalan there was a significant increase in tumour-cell kill. This effect was not observed with Sagatal or Hypnorm. Maximum increase in tumour-cell kill was achieved when Saffan was administered about 1 h before melphalan, and was dependent on Saffan dose. Clonogenic tumour-cell repopulation after melphalan was rapid (TD - 1 day) and the rate was similar from 2 levels of cell kill. When Saffan was combined with melphalan the repopulation rate was the same as with melphalan alone, and the increased cell kill was reflected in increased growth delay. The in vitro response of B16 melanoma cells to melphalan was unaltered by pretreatment with, or simultaneous exposure to Saffan. The results suggest that the mechanism of the enhanced cell kill in vivo is probably due to an indirect systemic effect, rather than a direct effect on the tumour cells.


Br. J. Cancer (1978) 38, 725

### INFLUENCE OF ANAESTHETICS ON TUMOUR-CELL KILL AND REPOPULATION IN B16 MELANOMA TREATED WITH MELPHALAN

J. H. PEACOCK AND T. C. STEPHENS

*From the Department of Radiotherapy Research, Institute of Cancer Research, Sutton, Surrey*

Received 1 August 1978 Accepted 28 August 1978

**Summary.—The influence of anaesthetics on the *in vivo* response of B16 melanoma to melphalan was studied using an *in vitro* cell-survival assay. Three anaesthetics were used, Saffan (Althesin) Sagatal (Nembutal) and Hypnorm. When Saffan was administered to tumour-bearing animals before melphalan there was a significant increase in tumour-cell kill. This effect was not observed with Sagatal or Hypnorm. Maximum increase in tumour-cell kill was achieved when Saffan was administered about 1 h before melphalan, and was dependent on Saffan dose.**

**Clonogenic tumour-cell repopulation after melphalan was rapid ($T_D = 1$ day) and the rate was similar from 2 levels of cell kill. When Saffan was combined with melphalan the repopulation rate was the same as with melphalan alone, and the increased cell kill was reflected in increased growth delay.**

**The *in vitro* response of B16 melanoma cells to melphalan was unaltered by pre-treatment with, or simultaneous exposure to Saffan. The results suggest that the mechanism of the enhanced cell kill *in vivo* is probably due to an indirect systemic effect, rather than a direct effect on the tumour cells.**

ANAESTHETICS are widely used in radiobiological studies on small animals, and sometimes in studies with cytotoxic drugs. However, it is becoming apparent that the use of anaesthetics for convenience is inadvisable, since they can produce major changes in blood pressure, tumour blood flow and body temperature (Johnson *et al.*, 1976) and can influence the response of normal and tumour tissue to cytotoxic treatments (Bruce *et al.*, 1970; Kaizer & Van Putten, 1973; Garattini *et al.*, 1974; Fu & Phillips, 1976). In this paper we describe the influence of several anaesthetics on the survival of B16 melanoma tumour cells to *in vivo* treatments with melphalan.

### MATERIALS AND METHODS

*Mice and tumours.*—Female C57BL mice were obtained from the Institute of Cancer Research breeding centre. They were used when 8–10 weeks old, and weighed 18–25 g.

B16 melanoma was obtained from the Roscoe B. Jackson Memorial Laboratory, Bar Harbour, Maine, U.S.A., in 1970. Tumours were transplanted s.c. into the flanks of mice, using the brei technique described by Stephens *et al.* (1977).

*Anaesthetics.*—A standard dose of each anaesthetic was chosen such that the mice remained unconscious for between 45 and 60 min. All anaesthetics were injected i.p. and as soon as the mice became unconscious they were transferred to an incubator at 35–36°C, which maintained their rectal temperature at $\sim$ 37°C.

Saffan (the veterinary equivalent of Althesin) was obtained from Glaxo Laboratories, Brentford, Middlesex. Each ml contains 9 mg alphaxalone (3α-hydroxy-5α-pregnane-11,20-dione) and 3 mg alphadalone acetate (21-acetoxy - 3α - hydroxy - 5α - pregnane - 11,20 - dione). The standard dose used was 90 mg/kg. Sagatal, the veterinary equivalent of Nembutal (pentobarbitone sodium) was obtained from May and Baker Ltd, Dagenham, Essex, and was used at a standard dose of

60 mg/kg. Hypnorm (fentanyl-fluanisone) a veterinary neurolept-analgesic manufactured by Janssen Pharmaceutica, Belgium, was obtained from Crown Chemical Company Ltd, Lamberhurst, Kent. The standard dose used was 10·2 mg/kg.

*Drugs.*—L-phenylalanine mustard (melphalan) was obtained from Burroughs Wellcome Ltd, Beckenham, Kent, in 100 mg vials. It was dissolved in acid-ethanol (1 ml) and the volume was made up to 10 ml with buffered diluent (both supplied by the manufacturers). For further dilutions PBS-"A" (Dulbecco & Vogt, 1954) was used.

*Preparation of cell suspensions.*—Cell suspensions were prepared as described previously (Stephens *et al.*, 1977). The mean cell yield per gram of tissue trypsinized for untreated tumours was $9{\cdot}9 \times 10^7$ (s.d. $2{\cdot}5 \times 10^7$, $n = 23$). Vital staining with trypan blue indicated viability > 95%.

In vitro *cell-survival assays.*—Survival of B16 melanoma cells was measured using the soft-agar assay first described by Courtenay (1976) and modified by Stephens *et al.* (1977). Viable cells varying in number from 500 to $2 \times 10^4$ were plated into 30 mm Petri dishes and the total cell number per dish was kept at $\sim 10^4$ by the addition of cells killed by irradiation (200 Gy). The culture medium used was Ham's F12 supplemented with 20% foetal calf serum (both supplied by Flow Laboratories Ltd, Irvine, Scotland). Cultures were incubated for 14–16 days at 37 °C in a water-saturated atmosphere of 5% $O_2$, 5% $CO_2$ in $N_2$.

Colonies of more than 50 cells were counted in at least 3 dishes per experimental point. The plating efficiency (PE) was calculated as the mean number of colonies per dish divided by the number of cells plated.

The mean PE of untreated B16 melanoma cells in this series of experiments was 0·47 (s.d. 0·16, $n = 23$). PEs as low as 0·0005 could be measured by this method. The cell kill in treated tumours was expressed either as surviving fraction (SF = PE treated/PE control) or as *fraction of surviving cells per tumour* (= SF × relative cell yield per gram × relative tumour weight). The former takes account only of changes in the colony-forming ability of the cells, whilst the latter also allows for changes in tumour size and cell yield.

In vitro *drug treatment.*—Cell suspensions of untreated tumours were prepared as described above, and the cells suspended in Ham's F12 culture medium. Drugs were added to the suspensions, and after 1 h of exposure the suspensions were centrifuged, the pellet washed and resuspended in fresh medium. Cell survival was measured in the soft-agar assay.

### RESULTS

*Influence of anaesthetics on melphalan dose–survival curve*

The dose–response curve of B16 melanoma to melphalan in conscious animals is shown in Fig. 1 (closed symbols). Assays were performed 18 h after treatment. The relationship between dose of melphalan and fraction of surviving cells per tumour is exponential, with a $D_{10}$ (the dose required to reduce cell survival to 10% of the control value) of 8 mg/kg.

Also shown in Fig. 1 is the survival curve for animals anaesthetized with Saffan

FIG. 1.—Dose–survival curves for B16 melanoma treated with melphalan either in conscious mice (●) or in mice anaesthetized with Saffan 20 min earlier (○).

FIG. 2.—Dose–survival curve for B16 melanoma treated with melphalan either in Sagatal - anaesthetized mice (▲) or Hypnorm-sedated mice (△). The solid line represents melphalan alone and the hatched line represents Saffan and melphalan, both from Fig. 1.

FIG. 3.—Dose–response curve for B16 melanoma treated with melphalan (7·5 mg/kg) after different doses of Saffan (○). Melphalan alone (●).

(90 mg/kg) 20 min before receiving melphalan (open symbols). Saffan alone had no effect upon the fraction of surviving cells per tumour, but did enhance the cell kill achieved with melphalan. The dose–response curve was again exponential but the $D_{10}$ was reduced to 4 mg/kg.

Fig. 2 shows the effect on the melphalan response curve of anaesthetizing animals with either Sagatal (closed symbols) or Hypnorm (open symbols) administered 20 min before melphalan. The solid line indicates the melphalan-alone curve and the broken line represents the Saffan + melphalan curve from Fig. 1. Pretreatment with these anaesthetics did not alter the tumour-cell kill with melphalan alone.

*Effect of Saffan dose*

Saffan was administered to tumour-bearing mice at various doses 20 min before melphalan (7·5 mg/kg). The dose–response curve is shown in Fig. 3. There is an enhanced effect, even at the lowest dose of Saffan (15 mg/kg), although this did not render the animals unconscious. At higher doses there is a further decrease in the number of surviving cells per tumour.

*Timing of Saffan administration*

Saffan (90 mg/kg) was administered at various times from 6 h before to 2 h after melphalan (7·5 mg/kg) and the results are shown in Fig. 4. In conscious animals this dose reduced survival to ~ 10% of the control. Maximum enhancement of melphalan cytotoxicity was achieved when Saffan was administered shortly before melphalan. Anaesthesia at this time approximately doubled the log kill of tumour cells due to melphalan alone.

When Saffan was given earlier, the degree of enhancement gradually fell and by 6 h no significant enhancement was apparent. Since animals only remained

FIG. 4.—Cell survival in B16 melanoma after treatment with Saffan (90 mg/kg) at various times before or after melphalan at 7·5 mg/kg (○). Melphalan alone (●).

FIG. 5.—Time-response curves for B16 melanoma treated with melphalan (7·5 mg/kg) alone (●), or with Saffan (90 mg/kg) administered 20 min before melphalan at 7·5 mg/kg (○).

unconscious for 45–60 min after this dose of Saffan, it seems that there was some enhancement even when melphalan was administered to mice which had regained consciousness. There was no enhanced cytotoxicity when Saffan was administered at times ranging from a few minutes to 3 h after melphalan.

*Duration of melphalan cytotoxicity*

Melphalan (7·5 mg/kg) was administered both to conscious animals and to animals anaesthetized with Saffan (90 mg/kg) 20 min earlier. Assays of cell survival were performed at various times later, and the results are shown in Fig. 5. Although there is considerable scatter in these data, the fitted lines suggest that in Saffan-pretreated mice the duration of melphalan cytotoxicity was increased by a factor of about 2. In both curves survival fell to a minimum and remained at that level for 24 h, suggesting that repair of melphalan damage does not occur.

*Repopulation studies*

The repopulation of B16 melanoma treated with melphalan alone and in combination with Saffan was examined by performing sequential cell-survival assays at various times after treatment. The growth of untreated tumours was not significantly different from that described previously (Stephens & Peacock, 1977). Tumour weight, total cells per tumour (tumour weight × cell yield per gram) and clonogenic cells per tumour (total cells per tumour × PE) all increased with a doubling time ($T_D$) of 3 days.

After treatment with melphalan at 7·5 mg/kg in unanaesthetized mice (Fig. 6a) tumour weight and total cells per tumour continued to increase, but with a $T_D$ of 3·5 days. Clonogenic cells per tumour were reduced by about one decade to $1{\cdot}5 \times 10^5$, and repopulation started immediately with a $T_D$ of 1 day.

After 15 mg/kg of melphalan, also in unanaesthetized mice (Fig. 6b), there was a greater reduction in the tumour growth ($T_D$ = 5 days) than at the lower dose. Total cells per tumour decreased in the first 3 days after treatment from $\sim 10^7$ to $3 \times 10^6$, and then recovered with a $T_D$ of 5 days. The total clonogenic cell number was reduced from $5 \times 10^6$ to $5 \times 10^3$ within 2 h of treatment. Re-

FIG. 6.—Cell repopulation curves for B16 melanoma treated with: (a) melphalan at 7·5 mg/kg, (b) melphalan at 15 mg/kg, and (c) Saffan (90 mg/kg) 20 min before melphalan at 7·5 mg/kg.

population began immediately with a $T_D$ of 0·8 days, until between Days 7 and 8, when the rate slowed to a $T_D$ of about 5 days, corresponding with that of the tumour weight and total cells per tumour.

When the lower dose of melphalan (7·5 mg/kg) was given to animals treated 20 min earlier with Saffan at 90 mg/kg (Fig. 6c), the initial cell kill and the pattern of repopulation were similar to those observed with the higher dose of melphalan alone (15 mg/kg).

*Influence of Saffan on melphalan dose–survival curve* in vitro

B16 melanoma cells were exposed *in vitro* to various doses of melphalan from 0·5 to 5 μg/ml (Fig. 7). Melphalan at 5 μg/ml produced 3 decades of cell kill. Cells were treated with Saffan (120 μg/ml) for 1 h, either immediately before or simultaneously with melphalan, but this did not increase the kill compared with melphalan alone. One hour's incubation of cells with this dose of Saffan alone did not change the PE.

### DISCUSSION

We have investigated the cytotoxic effects of melphalan in B16 melanoma, and have shown that enhanced cell kill occurs in animals which have been recently treated with the steroid anaesthetic Saffan. Anaesthesia *per se* did not appear to be essential, since some enhancement was seen with sub-anaesthetic doses of Saffan, and when melphalan was administered after the animals had regained consciousness after Saffan anaesthesia. No enhanced cell kill was seen when melphalan was administered to mice anaesthetized with Sagatal (a barbiturate hypnotic) or sedated with Hypnorm (a combined neuroleptic/analgesic). At concentrations estimated to be higher than would occur in animals, Saffan did not enhance the kill of B16 melanoma tumour cells by melphalan

FIG. 7.—*In vitro* dose-survival curve of B16 melanoma cells treated with melphalan alone (●), Saffan (120 μg/ml) followed by melphalan (▲) and Saffan (120 μg/ml) and melphalan administered simultaneously (○).

*in vitro*, implying that the agent did not directly affect cell sensitivity to melphalan.

The data in Fig. 5 suggest that there could be a doubling of the duration of melphalan cytotoxicity in Saffan-pretreated mice, and direct measurements of serum levels would be required to confirm this. A comparison of the potency of melphalan *in vivo* and *in vitro* suggests that it does not undergo any significant activation *in vivo*, so we can probably rule out altered activation of melphalan in Saffan-pretreated mice, and it is more likely that a reduced rate of melphalan clearance was responsible for the observed increase in cytotoxicity. There may be either slower metabolism to inactive products, or a reduction in the rate of excretion of the parent compound. Reduced body temperature is unlikely to be involved, since mice were maintained at 37°C by external heating when unconscious and during recovery.

The repopulation studies were performed to establish whether the enhanced cell kill achieved with melphalan + Saffan also led to enhanced growth delay, but they also allow a comparison of the repopulation after melphalan, with the repopulation patterns reported previously in the B16 melanoma for cyclophosphamide and CCNU (Stephens & Peacock, 1977). Our data suggest that Saffan was simply dose-modifying to melphalan, and that it enhanced growth delay when the agents were combined.

In our previous study we found that repopulation after CCNU was much faster than after cyclophosphamide. We have now shown that after treatment with melphalan, repopulation was similar to that after CCNU. We have also shown that the repopulation rate was not significantly different at 2 different levels of melphalan cell kill.

Whether there is any therapeutic advantage to be gained in tumour chemotherapy by combining the steroid Saffan with melphalan is not yet clear. In clinical chemotherapy other steroids, notably prednisone and prednisolone, are often included in regimes with melphalan. Experimentally, Wilkinson & Harrap (1978) have found increased antitumour effect when melphalan and prednisolone were combined. However, they also showed increased host toxicity due to the combination, and with melphalan + Saffan, Dr M. Y. Gordon (personal communication) has shown enhanced cell kill in mouse marrow cells (ADC-Cs) similar to that observed in B16 melanoma.

The results show clearly that Saffan anaesthesia markedly alters the response of both tumour and normal tissue to at least one cytotoxic treatment. Together with the work of others referred to above, our results stress the importance of including appropriate controls in all experiments, and reinforce the current trend of avoiding anaesthetics whenever possible.

We would like to thank Dr G. G. Steel for his support throughout this work and during the preparation of the manuscript.

### REFERENCES

BRUCE, D., LIN, H. & BRUCE, W. (1970) Reduction of colony forming cell sensitivity to arabinosyl cytosine by halothane anaesthesia. *Cancer Res.*, **30**, 1803.

COURTENAY, V. D. (1976) A soft agar colony assay for Lewis lung tumour and B16 melanoma taken directly from the mouse. *Br. J. Cancer*, **34**, 39.

DULBECCO, M. D. & VOGT, H. (1954) Plaque formation and isolation of pure lines with poliomyelitis viruses. *J. Exp. Med.*, **99**, 167.

FU, K. K. & PHILLIPS. T. (1976) The relative biological effectiveness (RBE) and oxygen enhancement ratio (OER) of neon ions for the EMT6 tumour system. *Radiology*, **120**, 439.

GARATTINI, S., BARTOSEK, I., DONELLI, M. G. & SPREAFICO, F. (1974) Interactions of anticancer agents with other drugs. In *Pharmacological Basis of Cancer Chemotherapy*. Baltimore: William and Wilkins, p. 565.

JOHNSON, R., FOWLER, J. F., & ZANELLI, G. D. (1976) Changes in mouse blood pressure, tumor blood flow, and core and tumor temperatures following nembutal or urethane anesthesia. *Radiology*, **118**, 697.

KAIZER, H. & VAN PUTTEN, L. (1973) The effect of sedative and anaesthetic drugs on killing of normal and malignant haemopoietic cells after chemotherapy. *Abst. 2nd meeting Eur. Assoc. Cancer Res.*, p. 186.

STEPHENS, T. C. & PEACOCK, J. H. (1977) Tumour volume response, initial cell kill and cellular repopulation in B16 melanoma treated with cyclophosphamide and 1-(2-chloroethyl)-3-cyclohexyl-1-nitrosourea. *Br. J. Cancer*, **36**, 313.

STEPHENS, T. C., PEACOCK, J. H. & STEEL, G. G. (1977) Cell survival in B16 melanoma after treatment with combinations of cytotoxic agents: lack of potentiation. *Br. J. Cancer* **36**, 84.

WILKINSON, R. & HARRAP, K. (1978) Modulation of alkylating agent toxicity and antitumour effect by steroids. *Br. J. Cancer*, **37**, 476.